# Effects of short-term fasting on cancer treatment

**DOI:** 10.1186/s13046-019-1189-9

**Published:** 2019-05-22

**Authors:** Stefanie de Groot, Hanno Pijl, Jacobus J. M. van der Hoeven, Judith R. Kroep

**Affiliations:** 1 Department of Medical Oncology, Leiden University Medical Center, Albinusdreef 2, P.O. Box 9600, 2300RC Leiden, The Netherlands; 2 Department of Endocrinology, Leiden University Medical Center, P.O. Box 9600, 2300RC, Leiden, The Netherlands

**Keywords:** Short-term fasting, Fasting-mimicking diet, Chemotherapy, Differential stress resistance, Differential stress sensitization, Toxicity

## Abstract

Growing preclinical evidence shows that short-term fasting (STF) protects from toxicity while enhancing the efficacy of a variety of chemotherapeutic agents in the treatment of various tumour types. STF reinforces stress resistance of healthy cells, while tumor cells become even more sensitive to toxins, perhaps through shortage of nutrients to satisfy their needs in the context of high proliferation rates and/or loss of flexibility to respond to extreme circumstances. In humans, STF may be a feasible approach to enhance the efficacy and tolerability of chemotherapy. Clinical research evaluating the potential of STF is in its infancy. This review focuses on the molecular background, current knowledge and clinical trials evaluating the effects of STF in cancer treatment. Preliminary data show that STF is safe, but challenging in cancer patients receiving chemotherapy. Ongoing clinical trials need to unravel if STF can also diminish toxicity and increase efficacy of chemotherapeutic regimes in daily practice.

## Background

Chronic caloric restriction reduces and delays cancer incidence, and inhibits tumor progression and metastasis in rodents [1–5]. Accordingly, cancer incidence and mortality are strongly reduced in chronic calorie restricted non-human primates [6]. Studies of long-term calorie restricted human subjects have shown a reduction of metabolic and hormonal factors associated with cancer risk [7–9]. However, chronic caloric restriction is not a feasible clinical intervention. Evident difficulties, such as the long period required to be effective, and unacceptable weight loss [10, 11], hamper clinical application in cancer patients.

Preclinical studies suggest that short-term fasting (STF) protects rodents from toxic effects of chemotherapy, while simultaneously enhances the efficacy of a variety of chemotherapeutic agents in numerous distinct malignancies, e.g. breast cancer, melanoma, neuroblastoma, pancreatic cancer, and colorectal cancer [12]. In distinct strains of mice bearing xenograft malignancies, tumor growth clearly slows down in response to chemotherapy combined with a 24–60 h fast as compared to treatment with chemotherapy alone [13–17]. STF simultaneously protects mice from chemotoxicity as well, because it reinforces stress resistance of healthy cells [17–24]. The distinct response of healthy versus tumor cells to STF is called differential stress resistance (DSR). During nutrient deprivation, healthy cells re-invest energy in maintenance and repair that contribute to resistance to chemotherapy, while tumor cells are unable to slow down growth due to mutations in tumor suppressor genes and mitogenic pathways [19, 25]. Moreover, low serum levels of glucose during STF impose extra stress on tumor cells, as their energy needs under these circumstances are primarily met by means of glycolysis [14]. As a consequence of these differential responses of healthy versus cancer cells to STF, chemotherapy causes more DNA damage and apoptosis in tumor cells, while leaving healthy cells unharmed when it is combined with STF. Thus, STF protects healthy cells against the toxic properties of chemotherapy and renders tumor cells more sensitive, a phenomenon called differential stress sensitization (DSS).

In contrast to most cancer therapies, STF has only mild side effects, such as headaches, dizziness, nausea, weakness and short-term weight loss in humans [26]. Therefore, STF is a promising strategy to enhance the efficacy and tolerability of chemotherapy in cancer patients, especially as STF is an affordable and accessible approach and is potentially effective in a wide variety of tumors [12]. However, patients with severe weight loss, sarcopenia, cachexia or malnutrition are probably not good candidates for a STF intervention [27, 28]. Recent guidelines recommend to increase protein and fat consumption in patients with cachexia [29, 30]. Thus, STF may be particularly useful for relatively fit patients treated with (neo)adjuvant chemotherapy.

This narrative review will cover the current knowledge of the molecular mechanisms explaining “differential stress resistance” of healthy- and cancer cells in response to STF. Moreover, it summarizes the available clinical data reflecting the impact of STF on the effects of chemotherapy in cancer patients. Finally, ongoing clinical studies of the effects of STF in cancer treatment will be critically reviewed.

## Differential stress resistance and sensitization in response to STF

**Fig. 1:**
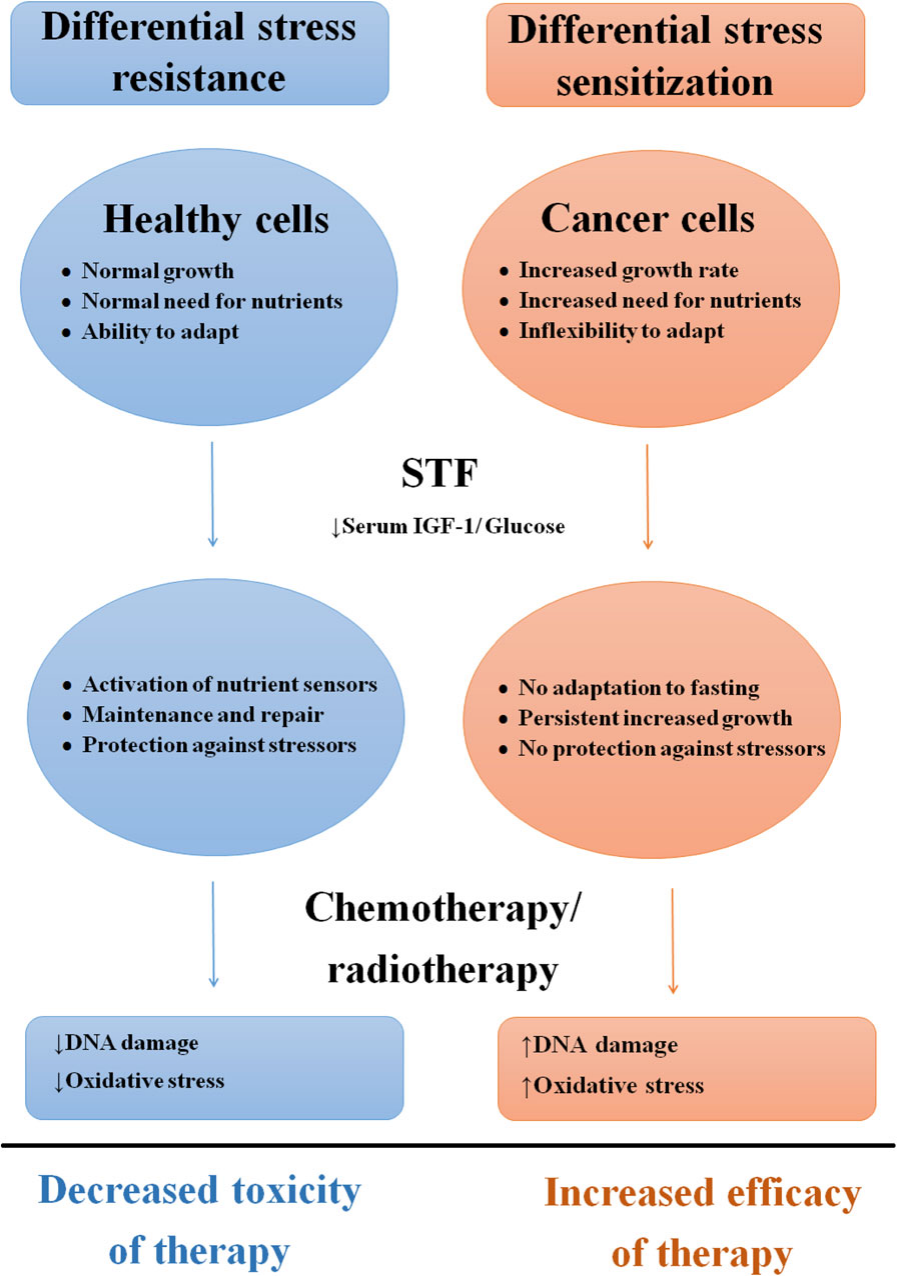
Schematic overview of differential effects of short-term fasting on healthy and cancer cells. Abbreviations: STF; short term fasting, IGF-1: insulin growth factor-1.

In healthy cells, nutrient deprivation shuts down pathways promoting growth to re-invest energy in maintenance and repair pathways (Fig. 1) [25, 31, 32]. This results in increased cellular protection, contributing to enhanced resistance to distinct stressors including chemotherapy and radiotherapy [19, 33]**.** In contrast, tumor cells are unable to activate this protective response, due to: 1) uncontrolled activation of growth pathways and self-sufficiency in growth signals caused by oncogenic mutations or autocrine production of growth factors, and 2) loss of anti-proliferative signals due to mutations in tumor suppressor genes [34]. Thus, acquiring the ability to increase growth, tumor cells lose the ability to adapt to extreme environments, including nutrient deprivation. Additionally, the persistent increased growth rate of tumor cells requires abundant nutrients [35]. Therefore, STF increases DSS of tumor cells to several chemotherapeutic agents, radiotherapy and tyrosine kinase inhibitors (TKIs) (Table 1) [12–16, 18–20, 36–40]. Although the exact mechanism of DSR and DSS by STF is unknown, several growth factors and nutrient sensing pathways have been proposed to be key regulators, of which insulin-like growth factor-1 (IGF-1) is the most examined [41–43]. Nutrient sensing pathways are activated or inhibited in response to a low amount of available nutrients and are highly conserved among distinct organisms to overcome periods of famine [44]. During nutrient scarcity, these pathways guide cells to invest energy in repair and maintenance rather than reproduction and growth [45–47], presumably to enhance survival of periods of famine. Analogously, infection-induced anorexia is a common sign of sickness and may be an important strategy for host defence [48, 49].

**Table 1:** Overview of in vivo studies of the effect of STF on the toxicity and/or efficacy of chemotherapy, radiotherapy and tyrosine kinase inhibitors

Author	Strain	Treatment	Outcomes of STF
Raffaghello et al. 2008 [19]	A/J, CD-1, nude mice and A/J mice bearing subcutaneous NXS2 neuroblastoma	High dose etoposide ± 48–60 h STF	Decreased mortality (toxicity) after high dose etoposide
Lee et al. 2012 [12]	BALB/c, C57BL/6 and nude mice bearing subcutaneous:	±48–60 h STF and:	
	4 T1 breast cancer	Cyclophosphamide	Increased efficacy of CT, STF alone was as effective as CT alone, Increased survival
	B16 melanoma	Doxorubicin	Increased efficacy of CT, Increased survival, Decreased metastasis
	GL26 glioma	Doxorubicin	Increased efficacy of CT STF alone was as effective as CT alone
	ACN human neuroblastoma	Doxorubicin	Increased efficacy of CT
	MDAMB-231 breast cancer	Doxorubicin	Increased efficacy of CT
	OVCAR3 ovarian cancer	Doxorubicin	Increased efficacy of CT
	NXS2 neuroblastoma	Only STF	STF alone was effective, Increased survival
Safdie et al. 2012 [13]	C57BL/6 N mice bearing subcutaneous or intracranial GL26 glioma	Temozolomide ± 48 h STF	Increased efficacy of CT, STF alone was as effective as CT alone (subcutaneous model only)
		Radiotherapy ± 48 h STF	Increased efficacy of radiotherapy
Shi et al. 2012 [37]	CD-1 Nude mice bearing subcutaneous ZL55 mesothelioma and A549 lung carcinoma	Cisplatin ±48 h STF	Increased efficacy of CT, STF alone was more effective as CT alone (mesothelioma only)
Kawaguchi et al. 2012 [62]	GFP-LC3 mice	Doxorubicin ±48 h STF	Decreased cardiotoxicity after high dose doxorubicin.
Brandhorst et al. 2013 [18]	AIN93G mice	High dose doxorubicin ±60 h STF	Decreased mortality (toxicity) after high dose doxorubicin.
Saleh et al. 2013 [38]	BALB/c mice bearing subcutaneous 67NR or NIH3 triple negative breast cancer	Radiotherapy ± 24 h STF (alternate)	Increased efficacy of radiotherapy
Cheng et al. 2014 [22]	C57BL/6 J mice	Cyclophosphamide ± 48 h STF	Decreased mortality (toxicity) after high dose cyclophosphamide.
Bianchi et al. 2015 [14]	BALB/c mice bearing subcutaneous CT26 colon cancer	Oxaliplatin ± 48 h STF	Increased efficacy of CT
Shim et al. 2015 [15]	C57BL/6 J mice bearing subcutaneous B16 melanoma	Doxorubicin or Cyclophosphamide ± 48 h STF	Increased efficacy of CT STF alone was as effective as CT alone
D’Aronzo et al. 2015 [36]	Nu/Nu mice bearing subcutaneous BxPC-3-luc pancreatic cancer	Gemcitabine ± 24 h STF	Increased efficacy of CT
Huisman et al. 2015 [20]	FabplCre;Apc15lox/C mice bearing spontaneous intestinal malignancies	Irinotecan ± 48 h STF	Decreased toxicity to CT No effect on efficacy of CT
Tinkum et al. 2015 [21]	B6(Cg)-Tyrc-2 J/J, Bmi1CreERT/+;Rosa26R/+ HopXCreERT/+;Rosa26R/+ Lgr5EGFP-IRES-CreERT2/+;Rosa26R/+, Lgr5EGFP-IRES-CreERT2/+ mice	High dose etoposide ± 24 h STF	Decreased mortality (toxicity) after high dose etoposide
Caffa et al. 2015 [39]	BALB/c mice bearing subcutaneous H3122 lung cancer or HCT116 colorectal cancer	Crizotinib/regorafinib ± 48 h STF	Increased efficacy of crizotinib/regorafinib
Huisman et al. 2015 [23]	BALB/c mice bearing subcutaneous C26 colon cancer	Irinotecan ± 72 h STF	Decreased toxicity to CT No effect on efficacy of CT
Di Biase et al. 2016 [16]	BALB/c, BALB/c-nude and C57BL/6 mice bearing subcutaneous MCF7 and 4T1 breast cancer, B16 melanoma	Doxorubicin and cyclophosphamide ±48 h STF or 96 h FMD	Increased efficacy of CT
Pietrocola et al. 2016 [40]	Wild-type C57BL/6 and athymic (nu/nu) mice	Mitoxantrone or oxaliplatin ± 48 h STF	Increased efficacy of CT
Di Biase et al. 2017 [17]	C57BL/6 mice	Doxorubicin ±24–72 h STF	Decreased cardiotoxicity after high dose doxorubicin.
Jongbloed et al. 2019 [24]	BALB/c mice	Irinotecan ± 72 h STF	Decreased toxicity to CT
Authors, site	Subjects	Treatment	Outcome of STF
Withers et al. 2014, UC Davis, USA [63]	20 dogs with lymphoma	Doxorubicin ±24 h STF	Safe and feasible Reduction in vomiting No reduction in IGF-1

*STF* Short-term fasting, *CT* Chemotherapy, *FMD* Fasting mimicking diet

### IGF-1 and insulin as key regulators of DSR

IGF-1 and insulin stimulate proliferation and growth and inhibit apoptosis in response to calorie and protein availability through signalling via the IGF-1 receptor (IGF-1R) and insulin receptor isoform A (IR-A), respectively [50–53]. Serum IGF-1 levels decrease during STF [54–56], because low insulin levels cause growth hormone (GH) resistance of the liver, which inhibits hepatic IGF-1 production [54, 57, 58]. Both insulin and IGF-1 activate the Ras/mitogen-activated protein kinase (MAPK) and phosphatidylinositol-3-kinase (PI3K)/Akt pathways. In healthy cells, inhibition of proliferation and/or investment in maintenance may contribute to increased stress resistance. For example, mice with a liver *Igf1* gene deletion (LID), which have decreased IGF-1 levels similar to those during STF [59–61], exhibit increased resistance to high doses of various chemotherapeutic agents [42] and this benefit was nullified through IGF-1 administration [12, 42]. Thus, the IGF-1R pathway seems to be a key mediator of stress resistance in response to STF in healthy cells.

During STF, the Ras/MAPK and PI3K/Akt pathways are down-regulated in cancer cells, whereby proliferation is inhibited [64]. Notably, resistance to the growth limiting effects of STF has been observed in cancer cells carrying mutations that cause a constitutive activation of the PI3K pathway, since these cells proliferate even in the absence of insulin or IGF-1 [65]. Therefore, the IGF-1R pathway is a key mediator of cancer cell growth and cancer resistance to commonly used therapeutics [42, 66, 67]. Thus, the reduction in circulating levels of IGF-1 and insulin during STF may contribute to the anticancer activity as well [68].

### AMPK and autophagy

AMP-activated protein kinase (AMPK) may play a major part in DSR due to STF [69]. AMPK monitors cellular energy levels and becomes activated when ADP:ATP or AMP:ATP ratios in the cell increase [70]. AMPK inhibits energy consuming processes, such as cell proliferation and protein synthesis, and activates energy generating processes, such as glycolysis and fatty acid oxidation. It inhibits cell growth and stimulates autophagy [71]. Autophagy (Greek for “self-eating”) is a highly conserved catabolic process among eukaryotes to survive periods of nutrient deprivation. This adaptive response of the cell involves damaged protein and organelle degradation to generate amino acids as an alternative energy source [72, 73]. Activation of AMPK and autophagy seems to play a major part in de protective effects of STF in healthy cells [17].

However, the effects of AMPK activation in distinct tumor cells may vary, as some tumors harbour constitutively active AMPK [74, 75] and others exhibit low AMPK activity [76–78]. Tumors with diminished AMPK activity or autophagy may be highly sensitive to STF, as AMPK activation enhances immune surveillance [40], whereas tumors with highly active AMPK or autophagy may be resistant [77, 79–82].

### Glucose metabolism and the “Warburg effect”

During STF, healthy cells, have the metabolic flexibility to cope with nutrient deprivation, since glucose can be replaced by ketone bodies and fatty acids as primary energy source.

In contrast, tumor cells depend on glucose to maintain the high rate of cellular proliferation [83, 84]. Akt stimulates the so called “Warburg effect”, characterized by an increased rate of glycolysis rather than oxidative phosphorylation even in the presence of oxygen [83–85]. STF down-regulates anaerobic glycolysis while up-regulating oxidative phosphorylation in tumor cells, and this “anti-Warburg effect” results in oxidative stress and apoptosis [14]. Also, a counterintuitive increase in protein translation during STF increases unmet energy needs, leading to cell death [12]. Moreover, the 20–40% reduction in circulating glucose during STF may be enough to kill anoxic tumor cells [85]. Thus, a decrease in nutrient availability during STF makes cancer cells more vulnerable to any challenge, including chemotherapy. However, overconsumption after a STF period might accelerate tumor growth, due to high glucose conditions and increased glycolysis [86].

### Reactive oxygen species and DNA damage

Chemotherapeutic agents inflict oxidative stress and DNA damage upon healthy cells, which are underlying mechanisms of toxicity [44, 87]. STF dampens oxidative stress in healthy cells by down-regulating metabolic rate and increasing scavenging of reactive oxygen species (ROS), which may contribute to DSR [33, 44]. As serum glucose levels decrease during STF, fatty acids serve as the main energy source. Beta-oxidation of fatty acids produces ketone bodies, which can be used as an alternative/additional fuel. Ketone bodies can also activate pathways involved in protection against ROS [88]. Moreover, STF presumably activates DNA repair processes in healthy cells [22]. For example, in mice fasted for 24 h before high dose infusion of etoposide, less DNA damage was seen in small intestinal stem cells 3 h after the infusion compared to mice who ate ad libitum. As 1.5 h post-treatment DNA damage was similar, DNA repair was likely more efficient in healthy cells due to STF [21].

In contrast, tumor cells exhibit increased ROS production if chemotherapy is combined with STF in vitro [12]. In breast cancer cells cultured in low glucose medium or serum of fasting mice, a 20-fold increase in DNA damage was seen in response to chemotherapy, as compared to cells cultured in regular medium or in serum of ad libitum fed mice [12].

### Immune competence

Chemotherapy causes bone marrow toxicity and depletion of circulating immune cells, especially myeloid cell depletion [89, 90]. Fasting protects hematopoietic stem cells and circulating immune cells from the detrimental effects of chemotherapy in mice [22, 91]. Additionally, more efficient immunity as a result of STF presumably causes a lower rate of infections and febrile neutropenia as well [92].

On the other hand, fasting improves the therapeutic effect of chemotherapy on the tumor possibly through cellular immunity in mice, as this effect is nullified in nu/nu mice, which lack T cells [40, 93]. Thus, STF may promote immunity and presentation of tumor-associated antigens (TAA), which promote efficient antitumor immunity contributing to increased efficacy of chemotherapy [94].

## From animal models to the clinic

Preclinical data documenting the benefits of STF is abundant and promising. However, words of caution are appropriate regarding its application in patients with cancer. For instance, preclinical studies show severe, albeit transient, weight loss in animal models (20–40% of total bodyweight after 24–48 h of fasting [12, 19, 23, 39, 95]. In contrast, the impact of a few days of fasting on bodyweight of humans appears far more modest (~ 1 kg per day, largely water loss) [26], which is probably explained by metabolic differences between humans and mice [95]. This is reassuring in the context of safety. However, it may also mean that humans need to fast for a (much) longer period of time than mice to obtain the same benefits (see discussion below). Therefore, carefully controlled clinical trials monitoring tumor growth as well as adverse effects of distinct dietary regimes are required before fasting mimicking diets (FMDs) can be applied in clinical practice.

## Metabolic risk factors for cancer

Obesity is associated with an increased risk of developing several cancers, such as breast cancer, colon cancer, ovarian cancer, endometrial cancer and thyroid cancer [96, 97] and IGF-1 levels are positively associated with the risk of developing breast and prostate cancer [98, 99]. Moreover, obesity and high levels of insulin and IGF-1, as well as having diabetes mellitus are associated with worse survival in cancer [100–103]. Obese subjects are often hyperglycemic and hyperinsulinemic, as a result of insulin resistance. Although circulating levels of total (free + bound) IGF-1 are normal or even low in obese subjects, levels of free (bioactive) IGF-1 are higher than in lean subjects [104]. Both insulin and free IGF-1 can bind the IGF-1R and IR-A [105] and activate the Ras/MAPK and PI3K/AKT pathway, through which cell proliferation is stimulated and apoptosis is inhibited, respectively [106]. Moreover, preclinically, obesity is associated with macrophage accumulation in adipose tissue resulting in an immune suppressive microenvironment [107]. These metabolic mechanisms may explain the increased risk of cancer as well as the worse prognosis of several cancers in obese subjects.

## Clinical studies of fasting

Voluntary fasting has been performed for many centuries and purposes, such as religious, ethical and cosmetic [26, 108]. Hippocrates was probably one of the first advocates of fasting for medical purposes (he recommended to fast during sickness). Since then, several doctors advised their patients to listen to their ‘fasting instinct’ (the natural loss of appetite during disease). Scientific research on the biomedical effects of fasting was performed from the late nineteenth century on, when several non-obese humans fasted for 20–40 days [26]. The first clinical study of medical fasting for the treatment of obesity was performed in 1915 [109]. The authors reported that short periods of four to six days of fasting is a safe and effective method for reducing bodyweight in obese humans. Since that time several studies were performed in obese subjects, with the longest fasting period lasting 382 days (!) [110, 111]. Fasting therapy was observed to be generally safe and well tolerated. Only mild side effects were reported, including headaches, dizziness, nausea, dyspepsia and fatigue [109–114]. However, in rare cases fasting for periods longer than 2 weeks was fatal in obese subjects with comorbidities as cardiac disease or diabetes mellitus [26, 115–117], and in one rare case a 53-day fast caused Wernicke encephalopathy in a patient with a lymphoma [118]. Additionally, fasting is not suitable for patients with rare metabolic illnesses such as glycogen storage disease or disorders of gluconeogenesis [119]. Benefits of fasting are improved cardiovascular risk factors, such as a decrease in blood pressure, improvement of lipid profile and insulin sensitivity, and weight loss in obese and non-obese subjects [114, 120]. The weight loss during STF is approximately 0.9 kg per day and decreases during prolonged fasting to 0.3 kg per day by the third week [26, 121]. Various studies examined the potential of fasting in the treatment of mood disorders, rheumatic diseases, asthma, chronic pain syndromes, hypertension, and metabolic syndrome [122, 123]. For example, a large cohort study of more than 2000 subjects with chronic illness and pain syndromes, who used a very low-calorie diet of 350 kcal per day for 7 days, showed an increase in quality of life without any serious side effect [122]. In healthy subjects, STF by 3 cycles of a fasting mimicking diet (FMD) reduces common risk factors for cardiovascular diseases, diabetes and ageing, such as lowering blood pressure, body weight, glucose, triglycerides and cholesterol [124]. Additionally, STF may improve clinical outcome in patients undergoing a partial liver resection and may prevent acute kidney injury after cardiac surgery [125, 126].

### Metabolic changes during STF in humans

STF has profound metabolic effects in humans [127]. Serum glucose levels drop after a few hours and are maintained at a lower level by endogenous glucose production, stimulated by glucagon. Glycogen storage capacity is limited so that stores are virtually depleted after 24 h. From then on, gluconeogenesis provides the brain with glucose as its major fuel source. Fatty acids are the primary fuel for the rest of the body. Beta-oxidation of fatty acids produces ketone bodies, which can serve as auxiliary energy source for the brain and the rest of the body. Insulin levels decrease rapidly and IGF-1 decreases dramatically after 36–72 h [41]. Since the liver is resistant to GH during prolonged fasting, IGF-1 production is profoundly reduced [128]. Diminished negative feedback control through reduction of circulating insulin and IGF-1 causes plasma GH levels to increase [129, 130]. IGF binding proteins, which regulate the bio-availability of IGF-1, change during fasting as well [41, 131, 132]. IGF-BP3 levels decrease, while IGF-BP1 levels increase 5–10-fold [133]. The decrease of IGF-I, downregulates the Ras/MAPK and PI3K/Akt pathways, through which cell proliferation is stimulated and apoptosis inhibited [12, 19]. Moreover, fasting down-regulates the hypothalamus-pituitary-thyroid axis activity. It particularly lowers triiodothyronine (T3), while thyroid stimulating hormone (TSH) and free thyroxine (fT4) are slightly decreased or not affected [134]. Clinical research shows that fasting periods longer than 48 h are required to facilitate a robust decrease in IGF-1 levels [41]. Therefore, it is likely that the positive effects of STF will be enhanced if the period of fasting is prolonged. A low sugar, low protein FMD may be an alternative to ease the burden of fasting, as it mimics the effects of STF on metabolism [91].

## Clinical studies of STF during chemotherapy

To date, a few small clinical studies in humans exploring the effects of STF combined with chemotherapy have been published (Table 2) [22, 131, 135–138]. The design and results of these studies in humans are summarized below.

**Table 2:** Overview of clinical studies on the effect of STF on the toxicity of chemotherapy

Authors, site	Human Subjects	Treatment	Outcome
Safdie et al. 2009, USC, USA [136]	10 human subjects with distinct malignancies	Distinct, + STF varying from 48 to 140 h prior and 5–56 h after CT	Safe and feasible. Reduction in CT-induced side effects.
Badar et al. 2014, KFMC, Saudi Arabia, NCT00757094 [135]	11 human subjects with distinct malignancies	IF during Ramadan when receiving CT	Safe and feasible. Reduction in CT-induced side effects^a^.
Dorff et al. 2016, USC, USA, NCT00936364, [22, 137]	20 human subjects with distinct malignancies	Platinum based CT + 24 h, 48 h or 72 h STF	Safe and feasible . Reduces DNA damage in leukocytes (dose response). Reduction of IGF-1 (dose response).
de Groot et al. 2015, LUMC, The Netherlands NCT01304251 [131]	13 women with stage II and III HER2 negative breast cancer	TAC CT ± 48 h STF	Safe and feasible. Reduction in IGF-1 Beneficial effect on erythrocytes and thrombocytes Possible reduction in DNA damage in healthy cells
Bauersfeld et al. 2018, Charite University, Germany, NCT01954836 [138]	34 women with breast and ovarian cancer	CT ± 60 h STF (cross-over)	Safe and feasible Beneficial effect on QOL, fatigue and well-being

*USC* University of Southern California, *KFMC* King Fahad Medical City, *LUMC* Leiden University Medical Center, *UC Davis* University of California, Davis School of Veterinary Medicine, *STF* Short-term fasting, *IF* intermittent fasting, *CT* Chemotherapy, *TAC* docetaxel/doxorubicin/cyclophosphamide, *IGF-1* insulin-like growth factor-1, *QOL* Quality of life  ^a^no statistics performed

In a case series from the University of Southern California (USC), 10 patients with distinct malignancies fasted in combination with docetaxel, carboplatin, paclitaxel and/or gemcitabine [136, 139]. Seven female and three male patients, with a median age of 61 years, diagnosed with breast (*N = 4*), prostate (*N = 2*), esophagus, non-small cell lung cancer, uterus and ovary cancer were described. Patients fasted for 48–140 h prior to, and 5–56 after commencing chemotherapy. Six of the ten patients fasted alternately during the chemotherapy cycles (the other four fasted every cycle) and side effects were compared between cycles combined with STF and chemotherapy alone. Side effects were scored according to the Common Terminology Criteria for Adverse Events (CTCAE) 4.0. Besides hunger and dizziness, fasting had no significant side effects. The authors reported a decrease in chemotherapy-induced side effects, including fatigue, weakness, vomiting and diarrhea, when chemotherapy was combined with STF compared to chemotherapy alone. In five patients the tumor volume (evaluated with PET or PET-CT) or tumor markers (PSA or CA-125) were evaluated. STF did not diminish chemotherapy-induced reduction of tumor volume and tumor markers, suggesting that STF did not interfere with the efficacy of chemotherapy.

In the King Fahad Medical City a clinical trial (NCT00757094) was conducted to evaluate the safety and feasibility of combining chemotherapy and intermittent fasting (including liquids) during the Ramadan [135]. Eleven patients, with distinct types of malignancies, received one gift of chemotherapy. Side effects and blood counts were compared with values measured in response to a similar dose of chemotherapy, given 2 weeks after the end of Ramadan. The authors concluded that combining fasting and chemotherapy during the month of Ramadan was well tolerated and safe. Side effects of chemotherapy tended to be less. However, because the study group was small, no statistics were performed. Moreover, due to the short fasting period (approximately 12 h), major benefits may not be expected, as IGF-1 levels will evidently not be reduced [140].

We performed a randomized pilot study (NCT01304251) to evaluate the effects of short-term fasting on tolerance to (neo) adjuvant chemotherapy in HER2-negative breast cancer patients in the Leiden University Medical Center (LUMC) [131]. Eligible patients had stage II/III breast cancer and received (neo)-adjuvant TAC (docetaxel/doxorubicin/cyclophosphamide) chemotherapy. Patients were randomized to fast 24 h before and 24 h after chemotherapy, or to eat according to the guidelines for healthy nutrition. Metabolic parameters (glucose, insulin and IGF-1) at baseline and immediately before chemotherapy infusion –when patients in the STF group had fasted for 24 h- were compared. Toxicity in the two groups was compared as well. Additionally, chemotherapy-induced DNA damage was quantified in peripheral blood mononuclear cells (PBMCs) by the level of γ-H2AX, as determined by flowcytometry. Thirteen patients were included, of whom seven were randomized to the STF arm. STF was well tolerated in our study. Plasma glucose levels increased and insulin levels remained constant in response to STF. We inferred that this phenomenon was the result of the concomitant use of dexamethasone, which was administered as an anti-emetic, for reduction of fluid retention and dampening of hypersensitivity reactions in response to docetaxel. Circulating IGF-1 levels were only modestly reduced in the study, which could be due to the use of dexamethasone as well [141, 142] or to the relatively short duration (24 h) of fasting prior to chemotherapy. Non-hematological toxicity did not differ between the groups. However, mean erythrocyte- and thrombocyte counts 7 days post-chemotherapy were significantly higher in the STF group compared to the non-STF group. Levels of γ-H2AX were significantly increased 30 min post-chemotherapy in CD45 + CD3- cells in non-STF, but not in STF patients [131]. This study provides evidence that STF attenuates bone marrow toxicity in these patients and reduces chemotherapy-induced DNA damage in PBMCs and/or accelerates its recovery.

Moreover, Dorff et al. reported results from a dose-escalating phase I study (NCT00936364), wherein 20 human subjects with distinct malignancies were treated with platinum-based chemotherapy combined with 24, 48 or 72 h STF to identify the optimal fasting duration [22, 137]. Eligible patients had distinct cancer types for which platinum-based combination chemotherapy was given with curative or palliative intent. Metabolic parameters (glucose, insulin, IGF-1 and IGF-BP1) at baseline and immediately before chemotherapy were compared. Moreover, toxicities and chemotherapy-induced DNA damage in PBMCs (determined by the COMET assay) between the three groups were compared. Twenty patients were included, 6 in the 24 h group and 7 in the 48 and 72 h group. The fasting was feasible and fasting-related toxicities were limited to grade 1 according CTCAE 4.0. The authors reported that 72 h of STF was associated with normal lymphocyte counts and maintenance of a normal lineage balance in white blood counts (lymphoid/myeloid ratio) after 2 cycles of chemotherapy, while 24 h STF was not [22]. IGF-1 levels decreased by 30, 33 and 8% in the 24, 48 and 72 h fasting cohorts, respectively, after the first fasting period. Additionally, the COMET assay showed reduced DNA damage 24 h after chemotherapy in leukocytes from subjects who fasted for more than 48 h compared with subjects fasted for 24 h *(P = 0.08).*

Finally, Bauerfeld et al. published a randomized cross-over trial (NCT01954836) evaluating the effect of STF on quality of life in breast cancer and ovarian cancer patients treated with chemotherapy [138]. Patients were randomized to fast, using an FMD, 36 h before and 24 h after chemotherapy or to eat a normocaloric Mediterranean diet for the first three cycles of chemotherapy. After three cycles the patient crossed over to the other group of nutrition (Mediterranean diet or fasting). The design of the study allows intra-individual comparisons regarding side effects of treatment, but precludes conclusions as efficacy of chemotherapy. In total, 50 patients were included in the study, but only 34 were analyzed because of early study discontinuation. The fasting was safe and feasible and five patients (14.7%) continued fasting after three cycles and did not cross over to the normocaloric diet. The authors concluded that STF led to a better tolerance to chemotherapy with less compromised quality of life (QOL) and reduced fatigue within the 8 days after chemotherapy. Moreover, 31 patients declared that they would fast again during chemotherapy, while only 3 patients declared that they would not fast again during chemotherapy.

These first clinical studies lack enough power to draw definite conclusions. However, the first results suggest that STF is safe, while it reduces toxicity of chemotherapy. Large scale randomized studies are required to get more insight in the benefits of STF in cancer treatment in humans.

## Ongoing studies

The first clinical studies have shown that STF combined with chemotherapy is safe and feasible in small patient groups [131, 136, 138]. Moreover, STF may reduce chemotherapy-induced toxicity. Additionally, chemotherapy-induced DNA damage in healthy cells may be decreased due to STF. However, large randomized clinical studies are required to generate (more) insight and validate the possible benefits of STF during chemotherapy. In Table 3, an overview is shown of the ongoing trials with STF combined with cancer treatment.

**Table 3:** Overview of ongoing or unpublished clinical trials of STF combined with chemotherapy or radiotherapy

Trial, site	*N*	Start	Tumor type and treatment,	STF	Primary endpoint
Non-randomized trial, NCT01175837, Mayo clinic, USA	12	2010	Distinct malignancies treated with CT	+ 24–48 h prior to chemotherapy (distinct regimens)	Safety and feasibility
Phase II randomized trial, NCT01802346, USC, USA	120	2013	Breast cancer treated with AC and prostate cancer treated with docetaxel	±96 h (using FMD) during CT	Toxicity of CT
Phase II/III Randomized study, NCT02126449, LUMC, the Netherlands	250	2014	Stage II and III Her2 negative breast cancer treated with AC-T or FEC-T.	±96 h (using FMD), during AC-T or FEC-T, no corticosteroids in control arm during AC or FEC	Phase II: toxicity of CT Phase III: pCR
Phase Ib non- randomized trial, NCT02379585, Western Regional Medical Center, USA	10	2015	Breast cancer	CT ± 48 h STF	pCR
Randomized trial, NCT02710721, Charite University, Berlin, Germany.	60	2016	Advanced metastatic prostate cancer	±60 h (using FMD) during CT vs. Mediterranean diet	QOL
Randomized crossover study, NTR5731, Erasmus medical center	18	2016	Metastatic colorectal cancer or other solid tumors receiving irinotecan	Dietary restriction including STF	25% reduction of the active irinotecan metabolite, SN38, in healthy liver tissue
Randomized trial, NCT03162289 Charite University, Berlin, Germany.	150	2017	Ovarian or breast cancer	±60 h (using FMD) during CT	QOL
Non-randomized trial, NCT03340935, University of Milan, Italy	85	2017	Distinct	5 days (using FMD), 700 kcal a day during cancer treatment	Toxicity of CT
Non-randomized trial, NCT03595540, Genova, Italy	60	2017	Distinct	5 days (using FMD), 700 kcal a day during cancer treatment	Feasibility
Randomized trial, NCT03709147, Milan, Italy	88	2018	Lung adenocarcinoma	± 5 days (using FMD) during CT in combination with metformin	PFS
Randomized trial. NCT03700437, Indiana University, USA	40	2018	Non-small cell lung cancer	± 96 h (using FMD) during carboplatin, pemetrexed and pembrolizumab	DNA damage in and count of circulating tumor cells
Studies of STF during radiotherapy					
Randomized trial, NCT01754350, Johann Wolfgang Goethe University Hospitals, Germany	50	2013	Glioblastoma Multiforme	±72 h during reirradiation	PFS

*USC* University of Southern California*, CT* Chemotherapy, *LUMC* Leiden University Medical Center, *AC-T* doxorubicin and cyclophosphamide followed by docetaxel, *FEC-T* 5-fluorouracil, epirubicin and cyclophosphamide followed by docetaxel, *FMD* Fasting mimicking diet, *pCR* pathological complete response, *QOL* Quality of life, *PFS* Progression-free survival

One study to date investigates the effects of STF on the effects radiotherapy. This randomized study (NCT01754350) conducted in Johann Wolfgang Goethe University Hospitals, includes patients with recurrent glioblastoma or gliosarcoma. The intervention entails 3 days of STF and 6 days of ketogenic diet during re-irradiation. The primary endpoint of the study is progression free survival.

A phase II study (NCT01802346), ongoing in the University of Southern California, examines the effects of an FMD on toxicity of chemotherapy in patients with breast and prostate cancer.

The phase II/III study (NCT02126449) from the LUMC, investigates the effects of STF using an FMD on toxicity (phase II part) and efficacy (phase III part) of neo-adjuvant AC-T or FEC-T chemotherapy. In this study prophylactic dexamethasone is omitted in the FMD arm during the AC and FEC chemotherapy cycles to reduce its potentially counteractive metabolic effects. Final results of the study are awaited [68]. The same FMD will be used to investigate the effect on circulating tumor cells in non-small cell lung cancer during treatment with carboplatin, pemetrexed and pembrolizumab.

Another FMD, described by Bauerfeld [138], is tested in two studies (NCT02710721, NCT03162289) conducted in the Charité University in Berlin, one in advanced metastatic prostate cancer and another in ovarian or breast cancer. Primary endpoint of both studies is QOL.

Finally, three studies (NCT03340935, NCT03595540 and NCT03709147) investigate the feasibility and effect of a 5-day FMD (approximately 700 kcal a day) on chemotherapy in distinct tumors and distinct chemotherapy regimens.

## Discussion and clinical implications

Clinical research evaluating the potential of STF is still in its infancy and more research is needed as the exact mechanism and effects are not established yet. Remaining questions are: is STF clinically effective in patients with solid tumors, in which tumors is STF effective, which markers are useful for prediction and monitoring of efficacy, what is the optimal length and timing of STF and refeeding, is STF safe in all patients, what is the optimal composition of an FMD, how can we increase patient’s compliance?

STF may be an affordable and safe intervention - at least in patients without severe weight loss or malnutrition -, which potentially dampens the side effects of chemotherapy, radiotherapy and TKI’s, while reinforcing their efficacy. Furthermore, it is potentially effective in a wide variety of tumors, although there is evidence that tumors with PI3K mutations or highly active AMPK are not sensitive [65, 82]. Reduction of side effects would improve quality of life and potentially reduce costs of hospitalization and the use of drugs such as anti-emetics or antibiotics. Moreover, STF may broaden the therapeutic window of cancer treatments, allowing for an increase of the dosage of (chemo) therapeutic agents, thereby enhancing their efficacy. However, STF might be only feasible in chemotherapeutic regimens characterized by: 1) bolus infusions on one day, to keep the fasting period short, 2) a long interval between two cycles, to ensure sufficient recovery time between cycles and 3) low dose or no use of corticosteroids, to avoid hyperglycemia, which might interfere with the benefits of STF [131].

Patients at risk for malnutrition or cachexia may not be candidates for STF, as it may be unsafe to further limit nutrient intake in these patients for even a short time [27]. However, notably, in preclinical setting caloric restriction showed even preservation of muscle strength in cancer cachexia [143]. Therefore, robust clinical trials are needed to establish the safety and efficacy of FMD in patients at high risk of cachexia.

Close monitoring of patients by nutritionists with expertise in fasting may be needed to increase compliance in future studies and to prevent patients unacceptable weight loss. Moreover, in our opinion, STF or FMDs should only be applied in the context of clinical research in patients with cancer until there is robust evidence for their safety and benefits.

## Conclusion

Abundant and convincing preclinical evidence shows that STF can decrease toxicity and simultaneously increase efficacy of a wide variety of chemotherapeutic agents. Preclinical data suggesting that STF can enhance the effects of radiotherapy and TKIs are promising as well. In clinical studies, STF emerges as a promising strategy to enhance the efficacy and tolerability of chemotherapy. It appears safe as an adjunct to chemotherapy in humans, and it may reduce side effects and DNA damage in healthy cells in response to chemotherapy. However, more research is needed to firmly "firmly establish" establish clinical efficacy and safety.

## Abbreviations

AMPK: AMP-activated protein kinase
CTCAE: Common Terminology Criteria for Adverse Events
DSR: differential stress resistance
DSS: differential stress sensitization
FMD: fasting mimicking diet
fT4: free thyroxine
GH: growth hormone
IGF-1: insulin-like growth factor-1
IGF-1R: IGF-1 receptor
IR-A: Insulin receptor isoform A
LID: liver *Igf1* gene deletion
LUMC: Leiden University Medical Center
MAPK: mitogen-activated protein kinase
PBMCs: peripheral blood mononuclear cells
PI3K: phosphatidylinositol-3-kinase
ROS: reactive oxygen species
STF: short-term fasting
T3: lowers triiodothyronine
TKIs: tyrosine kinase inhibitors
TSH: thyroid stimulating hormone
USC: University of Southern California
